# Assessing the Nursing Workload in the Cardiothoracic Intensive Care Unit: Comparative Study

**DOI:** 10.1002/nop2.70072

**Published:** 2024-10-30

**Authors:** Natasa Stojakovic, Aleksandra Matic, Andrej Preveden, Milenko Rosic, Milena Mikic, Vesna Rosic, Visnja Mihajlovic

**Affiliations:** ^1^ Institute of Cardiovascular Diseases of Vojvodina Sremska Kamenica Serbia; ^2^ Faculty of Medicine University of Novi Sad Serbia; ^3^ Institute for Child and Youth Health Care of Vojvodina Novi Sad Serbia; ^4^ Clinical Center of Vojvodina Novi Sad Serbia; ^5^ Department of Histology and Embryology, Faculty of Medical Sciences University of Kragujevac Kragujevac Serbia; ^6^ Department of Environmental Engineering, Technical Faculty Mihajlo Pupin in Zrenjanin University of Novi Sad Novi Sad Serbia

## Abstract

**Aims:**

This study aimed to assess nursing workload in Cardiac Intensive Care Unit (CICU) after three cardiothoracic surgery procedures during first four postoperative days using Nursing Activities Score (NAS) and Nine Equivalents of Nursing Manpower Use Score (NEMS) systems, to compare their performance for that purpose and to investigate association between nursing workload and type of surgery.

**Design:**

A comparative study.

**Methods:**

The research environment includes CICU of the University Hospital for Cardiovascular Diseases in Serbia. A total of 808 patients who underwent coronary, valvular, or combined surgery, resulting in 2282 filled NAS and NEMS pairs chart. Statistical analysis was performed using SPSS‐19. The correlation between NAS and NEMS was tested by Spearman's correlation coefficient. Differences were considered statistically significant at *p* < 0.05.

**Results:**

The lowest median of cumulative NAS 176 (175‐257) and NEMS 76 (64‐91) had coronary surgery patients, the highest NAS 224.5 (178‐334.5) and NEMS 83 (69‐121) had those with combined surgery; this difference was statistically significant (*p* < 0.001). The median of both scores decreased after surgery, with the following values from the first to the fourth postoperative day: NAS from 104 (102‐105) to 81 (74‐85) and NEMS from 46 (42–46) to 30 (30–37). The difference in mean values of both scores between the first and the fourth postoperative day was statistically significant (*p* < 0.001). NAS and NEMS were in a positive, strong correlation (*r* = 0.913; *p* < 0.005).

**Conclusion:**

Both scores can be used to measure nursing workload, identify the required number of nurses in CICU, and support task allocation. NAS may have an advantage because it better describes extensive postoperative monitoring and care needed for cardiac surgery patients. Nursing workload is associated with type of surgery, with the highest workload measured in patients who underwent combined surgery procedure and on the first postoperative day.

## 1 Background

Today, thanks to the continually improving surgical strategy and its supporting technology, cardiac surgery can be performed on an increasing number of high‐risk patients and those with complex health conditions. Nursing activities in the cardiothoracic intensive care unit (CICU) have a great impact on the overall outcome of patients who underwent different types of cardiothoracic surgery (Lee et al. 2017). Nursing activities are more and more complex and demanding in such patients, leading to increase in nursing workload (Lucchini et al. 2019; Crilly et al. 2019). The proper organisation of nurses in the intensive care unit (ICU) contributes to increased quality of postoperative care and reduces stress and nurse burnout (Moss et al. 2016; Bruyneel et al. 2021; Umutoni et al. 2019).

The optimal task allocation of nursing staff in every type of ICU, including cardiothoracic surgery‐oriented, is essential condition for high‐quality healthcare with minimum adverse events in critically ill patients (Riklikiene et al. 2020; Markewitz et al. 2020). Heavy nursing workload adversely affects patient safety and overall outcome in CICU. The incidence of healthcare‐associated infections (e.g., urinary tract infection, hospital‐acquired pneumonia) as well as the incidence of other complications (e.g., hemodynamic disturbances, inadequacy of mechanical ventilation after surgery) is increased (Carmona‐Monge, Perez, et al. 2013; Gomes et al. 2019; Silva et al. 2017). The higher incidence of different medical complications causes a higher risk of mortality and prolonged LOS in critically ill patients (Driscoll et al. 2018; Cyrino et al. 2018). Various types of adverse events, for example, fall from bed, infections related to central venous catheters and decubitus, are more frequent with the work overload of nurses, which also negatively affects patients' safety (Silva et al. 2017; Strazzieri‐Pulido et al. 2019; Carlesi et al. 2017). More complications and lower quality of overall healthcare lead to patient reduced satisfaction and increased medical costs (Ricci de Araújo et al. 2021; Oliveira et al. 2019). To allocate the tasks related to the patients' care in the most rational manner, objective assessment of nursing workload is necessary. This assessment should be informative and objective enough, without occupying too much working time for the performance (Gil et al. 2017; Greaves et al. 2018).

There are several instruments for such a purpose, out of which among the most widely accepted and used are the Nine Equivalents of Nursing Manpower Use Score (NEMS) (Miranda, Moreno, and Iapichino 1997) and the Nursing Activities Score (NAS) (Miranda et al. 2003). NEMS system was introduced in 1997 (Miranda, Moreno, and Iapichino 1997). It consists of just 9 items, so its scoring is not complicated nor time‐consumptive. NAS system is more complex than NEMS, having 23 items with sub‐items divided into 7 main categories of nursing activities. It expresses directly the percentage of working time of nursing staff during a wide range of activities in the care of a critically ill patient over 24 h period (Miranda et al. 2003).

According to studies for nursing workload assessment in ICU, NAS and NEMS are considered reliable workload assessment tools (Velozo et al. 2021; Vuković 2020; Carmona‐Monge, Rodríguez, et al. 2013; Macedo et al. 2016; Kraljic et al. 2017; Stafseth, Tønnessen, and Fagerström 2018). NAS indicates the workload of nurses, while NEMS, in addition to that, can be used to obtain additional information on the severity and prognosis of the disease, as well as to cost reduction (Carmona‐Monge, Rodríguez, et al. 2013; Macedo et al. 2016; Perren et al. 2014). Furthermore, in Switzerland NEMS is even a standard for calculating wages for nurses' work in ICU (Perren et al. 2014). While in Belgium, a widespread computerised systematic NAS encoding in hospitals is planned to be implemented in future (Bruyneel et al. 2018). NAS and NEMS are also used in the CICU to assess the workload of nurses (Perren et al. 2014; Hoogendoorn et al. 2020; Ducci, Zanei, and Whitaker 2008; Lucchini et al. 2014). NEMS is shorter and easier to assess, but less detailed, while NAS is more complicated and demanding to perform, but at the same time is more sensitive (Miranda et al. 2003; Carmona‐Monge, Rodríguez, et al. 2013).

The aim of this article was to assess nursing workload in CICU population after cardiothoracic surgery procedures using NAS and NEMS; to compare the instruments to determine which instrument is more accurate and best describes nursing workload in CICU following cardiothoracic surgery; and to explore the relation between nursing workload in CICU and type of cardiac surgery procedure.

## 2 Methods

### 2.1 Settings

This comparative study was conducted for the first time in the tertiary‐care university hospital (Clinic for Cardiovascular Surgery, Institute of Cardiovascular Diseases of Vojvodina, Serbia), with a 9‐bed CICU in 2019. The number of elected operations is about 100 per month, and it represents the workload of the unit.

### 2.2 Patients

All the patients of 18 years of age or older in which one of three operative methods (coronary, valvular or combined coronary‐valvular surgery) was performed and who spent at least 8 h after surgery in CICU were included in the study. All coronary surgery has been performed on‐pump. The above mentioned three types of cardiovascular surgery represent more than 90% of treatment modalities performed in our hospital. Therefore, patients with congenital heart defects, dissections of the aorta, heart tumours and some other heart and big vessel conditions, ECMO and VAD patients and urgent surgeries were excluded from the study. Losses were recorded during in‐hospital stay, but not during ICU stay. During this study, most of the patients have been discharged from CICU within 4 days. For that reason, 4 days of postoperative period was chosen as a time cut‐off point for the analysis in our study.

We observed that patients who underwent combined procedure required more nursing work than patients who underwent coronary procedure. We did a pilot study with 100 patients. Based on the obtained arithmetic means and standard deviations, the G Power calculator showed that a sample of at least 516 patients is required for NEMS and a sample of at least 744 patients for NAS. Our study lasted 10 months, from 1 January to 30 October, and included 808 patients.

### 2.3 Procedure

NEMS and NAS systems were used in their original form in English and translated to Serbian language by the authors of the study and back to English for checking. Both score translations were tested by nurses and health professionals who will administer the instrument and trialled for content and language clarity. For every examinee NEMS and NAS were calculated simultaneously, once per day, reporting preceding 24 h of nurse workload in CICU. Before the study started, 28 CICU nurses were educated and trained on using NEMS and NAS systems and their charts filling, as well as on recognising and linking their activities in the ICU with the parameters given in the tool. After the training, the nurses used NAS and NEMS in pilot study until the beginning of the study. The scoring procedure was supervised by CICU head nurse.

The nurses entered the activity data for 24 h into nursing documentation in the Hospital Information System (BIS), which was set to calculate the numerical values of both scores upon data entry. Average data‐entry time was 10–15 min. Data on the values of the NEMS and NAS systems (Appendix A: NEMS system, Appendix B: NAS system) were taken from the BIS. Variables have been analysed are NAS and NEMS parameters for assessment nursing workload. There are 23 parameters for NAS (monitoring and titration (1), laboratory (2), medication (3), hygiene procedures (4), care of all drains (5), mobilisation and positioning (6), support and care of relatives and patient (7), administrative and managerial tasks (8), respiratory support (9,10,11), cardiovascular support (12,13,14,15), renal support (16,17), neurological (18), metabolic support (19,20,21), specific intervention in and outside the ICU (22,23)) and 9 parameters for NEMS (basic monitoring (1), intravenous medication (2), mechanical ventilatory support (3), supplementary ventilatory care (4), supplementary ventilatory care (5), single vasoactive medication (6), multiple vasoactive medication (7), specific interventions in the ICU (8), specific interventions outside the ICU (9)).

The NEMS has a range between 0 and 56 points, a higher number of points indicates a greater involvement of the nurses, thus a more complex patient. One nurse can achieve an optimal 46 NEMS points during a shift (Carmona‐Monge, Rodríguez, et al. 2013; Perren et al. 2014). The NAS ranges from 1 to 177 points (Miranda et al. 2003; Stafseth, Solms, and Bredal 2011). NAS 50 points means that a nurse can work with two patients, NAS 100 means optimal nurse workload for 24 h and NAS above 100 requires additional number of nurses (Carmona‐Monge, Rodríguez, et al. 2013; Kraljic et al. 2017).

### 2.4 Statistical Data Analysis

Statistical analysis was performed using SPSS Version 19. The frequencies were expressed as absolute numbers and percentages. Continuous variables were presented as mean ± SD or median (25th percentile–75th percentile). Comparisons between the two groups were analysed by Mann–Whitney *U* test. Comparisons between more than two groups were done using the Kruskal‐Wallis test. The correlation between NAS and NEMS was tested by Spearman's correlation coefficient. Differences were considered statistically significant at *p* < 0.05.

### 2.5 Ethical Considerations

The study was approved by the Ethics Committee of the Institute for Cardiovascular Diseases of Vojvodina in Sremska Kamenica (decision no. 3055/1–8).

Patient consent was not required for this study, since the study was a prospective observational study without any invasive procedures. Respecting the principles of the Declaration of Helsinki, BIS data were collected prospectively and analysed retrospectively.

## 3 Results

In total, 808 examinees were included in the study, resulting in 2282 filled NAS and NEMS pairs chart (scores that were calculated on the same day for the same patients). All items in charts have been filled. The average age of 65.64 ± 8.45 years and predominantly male sex (66.6%) were observed in the study sample.

In 349 (43.1%) examinees coronary surgery was performed, in 263 (32.8%) valvular surgery, while 196 (24.2%) examinees underwent combined coronary‐valvular surgery. Length of stay (LOS) in CICU was the longest in examinees who underwent combined coronary‐valvular surgery and the shortest for those who had just coronary surgery. Furthermore, the examinees who had combined surgery had also the highest in‐hospital mortality. These data are shown in Table 1.

**TABLE 1:** Data about age, sex, LOS* in the cardiothoracic intensive care unit and in‐hospital mortality in the study group in total and according to the type of surgery.

Type of surgery	No (%)	Average age (±SD)	Male sex (No, %)	LOS* in CICU** (Days median***)	In‐hospital mortality (No, %)
Coronary	349 (43.1%)	64.39 ± 7.99	248 (71.1%)	0.98 (0.85–1.79)	4 (1.1%)
Valvular	263 (32.8%)	65.34 ± 8.84	158 (60.1%)	1.78 (0.96–2,79)	11 (4.2%)
Coronary‐valvular	196 (24.2%)	68.35 ± 8.17	132 (67.3%)	2.86 (1.82–5.24)	25 (12.8%)
Total	808 (100%)	65.64 ± 8.45	538 (66.6%)	1.19 (0.94–2.81)	40 (5.0%)

* Length of Stay.

** Cardiothoracic Intensive Care Unit.

*** Median (25th percentile–75th percentile).

According to the surgery type, the cumulative mean values of NAS, as well as its median and 25th and 75th interquartile range, were the highest in the patients who underwent combined coronary‐valvular surgery and the lowest after coronary surgery. The same stands for cumulative mean value, median and 25th and 75th interquartile range of NEMS. Comparison of cumulative mean values of NAS and NEMS between the types of surgery was statistical significant. These results are displayed in Table 2.

**TABLE 2:** Cumulative mean value of NAS* and NEMS** in examinees in total study sample and from each group according to the type of surgery.

Type of surgery	NAS*						NEMS**					
	MEAN	SD***	25th	50th (median)	75th	*p*	MEAN	SD***	25th	50th (median)	75th	*p*
Coronary	229.50	145.86	175.0	176.0	257.0	< 0.005	87.81	61.07	64.0	76.0	91.0	< 0.005
Valvular	250.94	131.13	176.0	179.0	265.5		97.75	57.67	64.0	76.0	112.0	
Coronary‐valvular	281.23	194.45	178.0	224.5	334.5		111.26	84.28	69.0	83.0	121.0	

*Note:* Statistical significance at *p* < 0.05.

* Nursing Activities Score.

** Nine Equivalents of Nursing Manpower Use Score.

*** Standard Deviation.

Comparison of cumulative mean values of NAS and NEMS between the pairs of types of surgery showed significant differences according to these parameters. The lowest NAS, as well as NEMS, were in the coronary group, while both scores showed the highest values in the coronary‐valvular group, Table 2. Comparison of coronary surgery with valvular as well as with combined surgery showed high statistical significance of the difference, while this difference was less pronounced when valvular and combined surgery were compared. This was observed with both NAS and NEMS. These results are shown in Table 3.

**TABLE 3:** Comparison of cumulative mean values of NAS* and NEMS** between the pairs of groups according to the type of cardiothoracic surgery, as well as between all the three groups.

Type of cardiothoracic surgery	NAS*	NEMS**
Coronary vs. valvular	*p* < 0.005	*p* < 0.005
Coronary vs. coronary‐valvular	*p* < 0.005	*p* < 0.005
Valvular vs. coronary‐valvular	*p* = 0.019	*p* = 0.038
Difference between the groups	*p* < 0.005	*p* < 0.005

*Note:* Statistical significance at *p* < 0.05.

* Nursing Activities Score.

** Nine Equivalents of Nursing Manpower Use Score.

**FIGURE 1:**
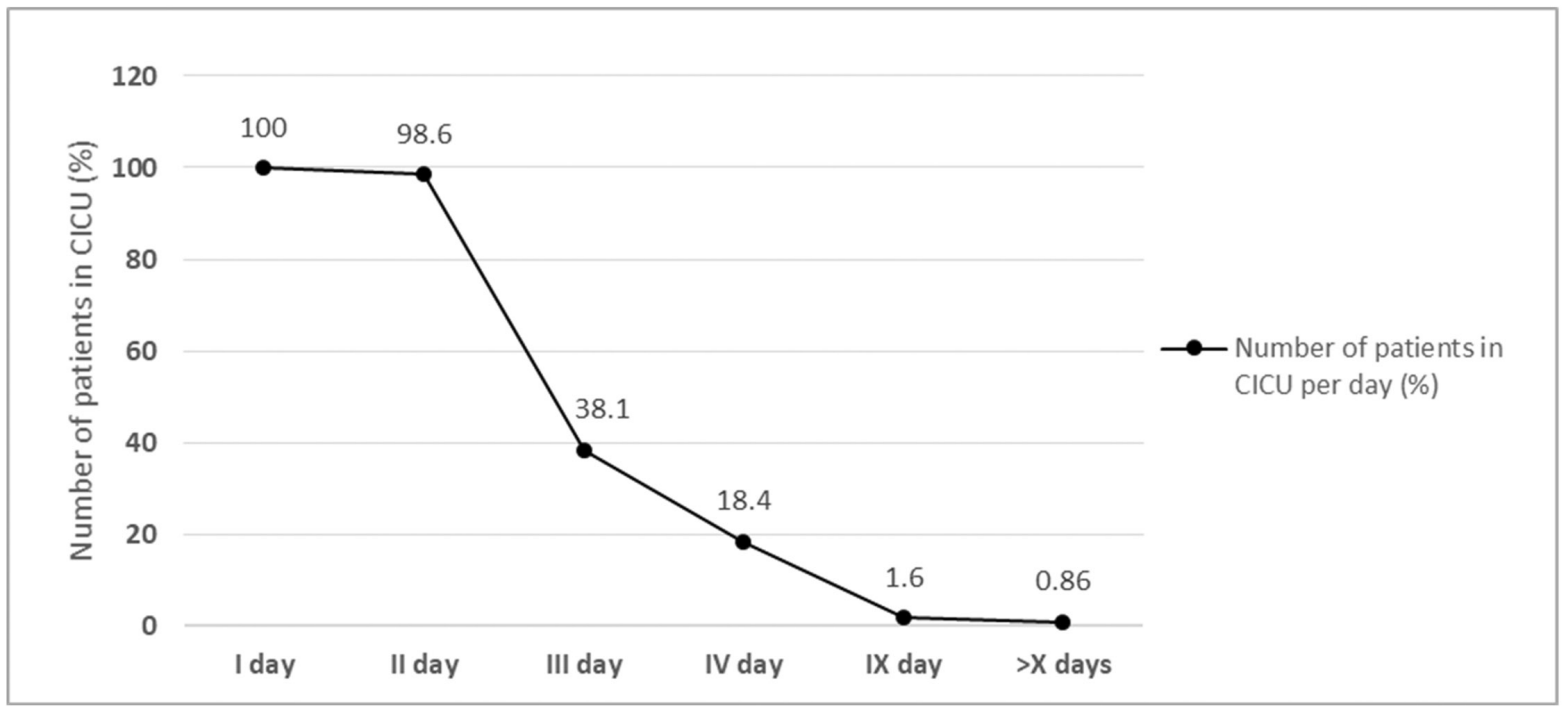
Distribution of percentage of examinees according to postoperative days spent in the cardiothoracic intensive care unit (CICU)*. * Cardiothoracic Intensive Care Unit (CICU).

The following Figure 1 shows the length of stay in the cardiothoracic intensive care unit in the form of percentages of patients who spent 1 day, 2 days, etc. in the cardiothoracic intensive care, Figure 1.

NAS and NEMS were performed on a daily basis, and mean values of both scores for each of the four postoperative days in CICU were analysed. Mean values and medians of NAS and NEMS during postoperative Day 1 were obviously higher compared to postoperative Days 2, 3 and 4. Moreover, postoperative Days 2, 3 and 4 had rather similar mean and median values of the scores. Data about NAS and NEMS per day during the first four postoperative days in CICU are shown in Table 4.

**TABLE 4:** Comparison of mean, standard deviation, median and 25th and 75th interquartile values of NAS* and NEMS** during the first four postoperative days.

Post‐operative day	No of examinees	NAS*						NEMS**					
		MEAN	SD***	25th	50th (median)	75th	*p*	MEAN	SD***	25th	50th (median)	75th	*p*
0ne		102.94	4.01	102.0	104.0	105.0	< 0.001	42.59	6.50	42.0	46.0	46.0	< 0.001
Two	798	77.87	6.20	82.0	83.0	87.0		28.18	8.21	30.0	35.0	39.0	
Three	308	81.26	9.22	82.0	83.0	87.0		30.19	7.63	30.0	30.0	37.0	
Four	149	82.04	10.27	74.0	81.0	85.0		31.43	6.99	30.0	30.0	37.0	

*Note:* Statistical significance at *p* < 0.05.

* Nursing Activities Score.

** Nine Equivalents of Nursing Manpower Use Score.

*** Standard Deviation.

For total study sample, mean value of NAS was 89.99 ± 8.50, while mean value of NEMS was 39.71 ± 11.08 per CICU day, Table 5.

**TABLE 5:** Daily NAS* and NEMS** values for total study sample.

	Minimum	Maximum	MEAN	SD***
NAS*	77.87	108.50	89.990	± 8.500
NEMS***	28.18	81.21	39.706	± 11.075

* Nursing Activities Score.

** Nine Equivalents of Nursing Manpower Use Score.

*** Standard Deviation.

**FIGURE 2:**
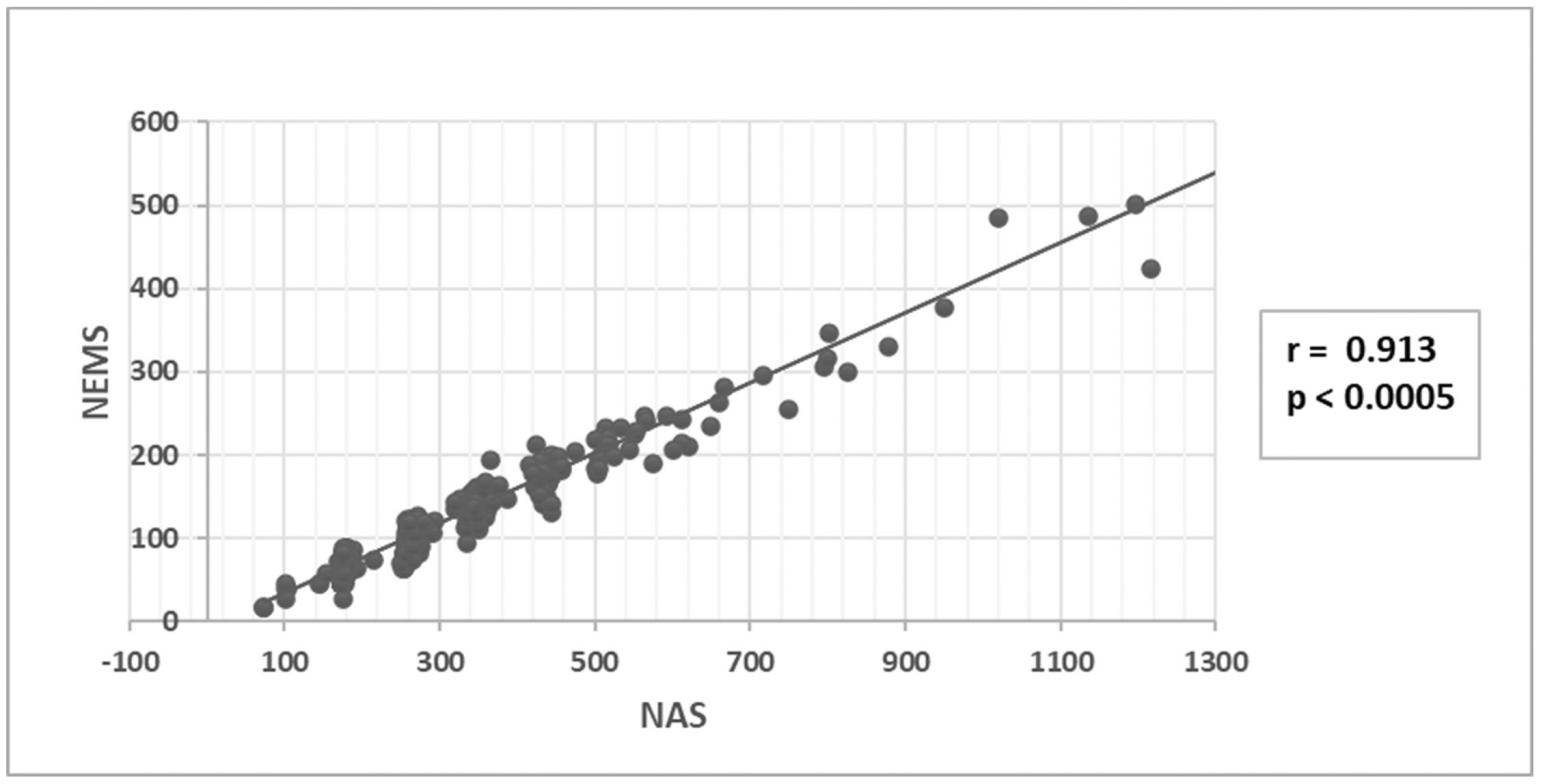
Correlation of NAS* and NEMS**. *Note:* Statistical significance at *p* < 0.05. * Nursing Activities Score. ** Nine Equivalents of Nursing Manpower Use Score.

Spearman's correlation test showed a positive correlation of NAS and NEMS in our study sample, and Spearman's coefficient value of 0.913 showed that this correlation is very strong, Figure 2.

## 4 Discussion

When one wants to compare nurses' workload in different settings, it is crucial to make comparisons between similar healthcare facilities. The results for NEMS and NAS in the literature for different cardiothoracic intensive care units are not the same. Ducci et al. conducted the study in highly specialised CICU in which mean value of NEMS was 59.7, while mean value of NAS was 73.7 (Ducci, Zanei, and Whitaker 2008). Mean value of NAS measured in Italian CICU was 63.51 ± 14.69 (Lucchini et al. 2014). In another study done in Italian polyvalent ICU, mean NAS and NEMS were 76.12 ± 14.66, 32.05 ± 2.11, respectively, while highest value for NAS for extracorporeal circulation 102.26 ± 5.70 (37). Authors from Norway got mean NAS value of 96.24 ± 22.35 (Stafseth, Solms, and Bredal 2011). The mean value of NEMS in our study was 39.71 ± 11.08, while mean value of NAS was 89.99 ± 8.50.

In our study, NEMS and NAS mean values are higher compared to the above‐mentioned studies. According to our open‐heart surgery procedure with extracorporeal circulation, patients on arrival at the ICU need 24‐h monitoring (i.e., ventilatory, cardiovascular, renal, neurological and metabolic support) that consists of continuous monitoring, recording and registration of irregularities. For these reasons, we believe that the values of NAS and NEMS in our study are higher compared to the above studies. However, high values of NAS in Norway study are related to fact time spent with families of patients (Stafseth, Solms, and Bredal 2011). High value for NAS in Italian polyvalent ICU (Lucchini et al. 2008) and high value NAS for first postoperative day in our study are due to patients who were on extracorporeal circulation.

High workload (Inadequate N/P ratio) can affect the frequency of intrahospital infections as well as other complications, higher risk of mortality and prolonged bed rest, also leaves less time for nurses to cooperate with doctors, which negatively affects synchronised and most effective teamwork in performing various tasks of modern healthcare. More complications and lower quality of overall healthcare affect patient safety and lead to reduced patient satisfaction.

Many levels of organisation are of great influence on nurses' heavy workload. Furthermore, the workload changes from 1 day to another, sometimes quite substantially. Patient's demands and healthcare depends on postoperative day—on day 1 NEMS and NAS were significantly higher compared to rest of postoperative days. In our study, NAS on day one was 102.95 ± 4.01, while for the next 3 days were lower. In addition, the study conducted in CICU in Brazil (Dias 2006) got also the highest NAS score on the first postoperative day, which was 96.79. Several studies confirmed a significant difference in nurses' workload in every CICU patient depending on postoperative day (Kraljic et al. 2017; Peng, Mayner, and Wang 2014).

Our study results showed that our patients during the last 24 h in ICU have not presented high level of nursing workload, Table 4. Administrative and management activities, patient discharge procedure, do not have high nursing scores, because according to hospital procedure nurses from medium care unit transfer patients discharged from the ICU. Therefore, it has not presented high level of nursing workload, as in Sardo et al. (2023).

Some CICUs might be specialised in certain types of cardiothoracic surgery and therefore have different profiles of patients, which has also a great impact on patients' healthcare demands (Kraljic et al. 2017). Our results show a significant difference in NEMS and NAS scores depending on which type of cardiothoracic surgery was performed. According to the results related to the type of surgery, the obtained total values of NAS and NEMS for combined surgery are (were) higher than those calculated for the other two types of surgery, which corresponds to the severity of patient's condition following a combined surgical procedure. In addition, combined patients had three times longer LOS, 2.86. Therefore, when planning the operative programme and the optimal number of nurses during the postoperative period in the ICU, it is necessary to take into consideration the type of operation as a factor that affects the workload of nurses (Riklikiene et al. 2020).

Some similar studies to assess nursing workload in ICU using NAS and NEMS showed low correlation between NEMS and NAS (Stafseth, Solms, and Bredal 2011; Adell et al. 2006), while some studies showed high correlation between two tools (Velozo et al. 2021; Carmona‐Monge, Rodríguez, et al. 2013; Ducci, Zanei, and Whitaker 2008). Our results showed also a high positive correlation between the two score systems, with Spearman's coefficient of 0.913. Therefore, both scales can be used to measure nurse workload in our ICU. However, NAS consists of higher number of nursing activities based on independent activities of nursing care, compared to NEMS, which is based only on therapeutic interventions to critically ill patients (Carmona‐Monge, Rodríguez, et al. 2013). Despite the high correlation between NAS and NEMS, an important factor in choosing a score is certainly the time needed to complete its chart. Due to the number of activities to be entered, NEMS certainly requires shorter completion time compared to NAS, while on the other hand, NAS covers 81% of nursing activities (Miranda et al. 2003; Carmona‐Monge, Rodríguez, et al. 2013; Hoogendoorn et al. 2020). Therefore, NEMS does not capture specific information of nurse workload, which provides a measure of nursing workload derived from direct patient care, which is equally important to us for evaluation of nursing work.

Several research studies show that NAS does not only have the best overall performance in measuring nursing workload but, since it makes nursing workload visible, it is also effective in measuring nursing staff costs (Velozo et al. 2021; Hoogendoorn et al. 2020; Bruyneel et al. 2022).

## 5 Limitations

This study is based on data from a single cardiac surgery centre, and in addition, as the number of prior research on this study topic is not large, it can be associated with higher risk of limited and biased results and with potential limits to the generalisability, extrapolation of the results to other centres and institutions. Secondly, this study did not include operative risk factors defined in EuroSCORE II, which could affect the size of correlation between patient's operative risks and nurses' workload after surgery.

## 6 Implications

Both NAS and NEMS can be used to measure nursing workload in CICU and allow to predict the intensity of nursing activities in similar settings. Both systems can facilitate planning of the required number of nurses and optimal task allocation between the nursing staff, as well as contribute to increasing patient safety. Type of the cardiac surgery procedure can be used as a valid indicator for predicting the nursing workload in CICU and for planning the adequate nursing workload. In patients with combined coronary‐valvular surgery, it is necessary to anticipate larger number of nurses in CICU after the operation. The nursing workload is the highest during the first postoperative day.

## 7 Conclusions

This study represents one of the first studies carried out in Serbia, in Vojvodina region in the Institute of Cardiovascular Diseases, Clinic for Cardiovascular Surgery. This research could be a base for future similar studies at the national level related to the assessment of the nursing workload in ICUs of different levels and typologies. Results show that both scores can be used to measure nursing workload. NAS may have an advantage because it better describes extensive postoperative monitoring and care needed for cardiac surgery patients, and it contains more criteria than NEMS and describes in more detail the nursing workload in cardiothoracic surgery patients.

Proper sizing of the nursing team at appropriate times, based on the type of cardiac surgery procedure and realistic expectations of the severity of postoperative recovery in each CICU can lead to significant rationalisation and optimization of human resources, while improving patient safety, patient outcomes and quality of care.

Also, results showed that the highest nursing workload can be expected during care of the patients in CICU who underwent combined coronary‐valvular surgery and during the first postoperative day.

## Author Contributions

**Natasa Stojakovic:** investigation, writing – original draft preparation. **Aleksandra Matic:** methodology, formal analysis. **Andrej Preveden:** writing – review and editing, data curation. **Milenko Rosic:** supervision; **Milena Mikic:** conceptualisation, writing – original draft preparation. **Vesna Rosic:** data curation, supervision. **Visnja Mihajlovic:** validation; writing – review and editing.

## Conflicts of Interest

The authors declare no conflicts of interest.

## Data Availability

Author elects to not share data.

## Appendix A Nine Equivalents of Nursing Manpower use Score (NEMS)

### A.1

**Table:**

Nine Equivalents of Nursing Manpower Use Score			
	Item		Points
1	Basic monitoring	Hourly vital signs, regular record and calculation of fluid balance	9
2	Intravenous medication	Bolus or continuously, not including vasoactive drugs	6
3	Mechanical ventilatory support	Any form of mechanical/assisted ventilation, with or without PEEP (e.g., continuous positive airway pressure), with or without muscle relaxants	12
4	Supplementary ventilatory care	Breathing spontaneously through endotracheal tube; supplementary oxygen any method, except if (3) applies	3
5	Single vasoactive medication	Any vasoactive drug	7
6	Multiple vasoactive medication	More than one vasoactive drug, regardless of type and dose	12
7	Dialysis techniques	All	6
8	Specific interventions in the ICU	Such as endotracheal intubation, introduction of pacemaker, cardioversion, endoscopy, emergency operation in the past 24 h, gastric lavage; routine interventions such as X‐rays, echocardiography, electrocardiography, dressings, introduction of venous or arterial lines, are not included	5
9	Specific interventions outside the ICU	Such as surgical intervention or diagnostic procedure; the intervention/procedure is related to the severity of illness of the patient and makes an extra demand upon manpower efforts in the ICU	6

## Appendix B Nursing Activities Score (NAS)

### B.1

**Table:**

Nursing Activities Score		
	Item	Points
1	Monitoring and titration	
1a	Hourly vital signs, regular registration and calculation of fluid balance	4.5
1b	Present at bedside and continuous observation *or* active for 2 h or more in any shift for reasons of safety, severity or therapy, such as non‐invasive mechanical ventilation, weaning procedures, restlessness, mental disorientation, prone position donation procedures, preparation and administration of fluids and/or medication, assisting specific procedures	12.1
1c	Present at bedside *and* active for 4 h or more in any shift for reasons of safety, severity or therapy such as those examples above (1b)	19.6
2	Laboratory biochemical and microbiological investigations	4.3
3	Medication, vasoactive drugs excluded	5.6
4	Hygiene procedures	
4a	Performing hygiene procedures such as dressing of wounds and intravascular catheters, changing linen, washing patient, incontinence, vomiting, burns, leaking wounds, complex surgical dressing with irrigation, special procedures (e.g., barrier nursing, cross‐infection related, room cleaning following infections, staff hygiene), etc.	4.1
4b	The performance of hygiene procedures took > 2 h in any shift.	16.5
4c	The performance of hygiene procedures took > 4 h in any shift	20
5	Care of all drains, except gastric tube	1.8
6	Mobilisation and positioning, including procedures such as turning the patient, mobilisation of the patient moving from bed to chair and team lifting (e.g., immobile patient, traction, prone position)	
6a	Performing procedure(s) up to three times per 24 h	5.5
6b	Performing procedure(s) more frequently than three times per 24 h or with 2 nurses	12.4
6c	Performing procedure with three or more nurses, any frequency	17
7	**Support and care** of relatives and patient, including procedures such as telephone calls, interviews, counselling; often, the support and care of either relatives or patient allow staff to continue with other nursing activities (e.g., communication with patients during hygiene procedures, communication with relatives while present at bedside and observing patient)	
7a	Support and care of either relatives or patient requiring full dedication for about 1 h in any shift such as to explain clinical condition, dealing with pain and distress, difficult family circumstances	4
7b	Support and care of either relatives or patient requiring full dedication for 3 h or more in any shift such as death, demanding circumstances (e.g., large number of relatives, language problems, hostile relatives)	32
8	Administrative and managerial tasks	
8a	Performing routine tasks such as processing of clinical data, ordering examinations, professional exchange of information (e.g., ward rounds)	4.2
8b	Performing administrative and managerial tasks requiring full dedication for about 2 h in any shift such as research activities, protocols in use, admission and discharge procedures	23.2
8c	Performing administrative and managerial tasks requiring full dedication for about 4 h or more of the time in any shift such as death and organ donation procedures, coordination with other disciplines	30
9	Respiratory support: Any form of mechanical ventilation/assisted ventilation with or without positive end‐expiratory pressure, with or without muscle relaxants; spontaneous breathing with positive end‐expiratory pressure (e.g., CPAP or biphasic positive airway pressure [biPAP]), with or without endotracheal tube; supplementary oxygen by any method	1.4
10	Care of artificial airways: endotracheal tube or tracheostomy cannula	1.8
11	Treatment for improving lung function: thorax physiotherapy, incentive spirometry, inhalation therapy, intratracheal suctioning	4.4
12	Vasoactive medication, disregard type and dose	1.2
13	Intravenous replacement of large fluid losses. Fluid administration > 3 L/m^2^/day, irrespective of type of fluid administered	2.5
14	Left atrium monitoring: pulmonary artery catheter with or without cardiac output measurement	1.7
15	Cardiopulmonary resuscitation after arrest, in the past period of 24 h (single precordial thump not included)	7.1
16	Hemofiltration techniques, dialysis techniques	7.7
17	Quantitative urine output measurement (e.g., by indwelling urinary catheter)	7
18	Measurement of intracranial pressure	1.6
19	Treatment of complicated metabolic acidosis/alkalosis	1.3
20	Intravenous hyperalimentation	2.8
21	Enteral feeding through gastric tube or other gastrointestinal route (e.g., jejunostomy)	1.3
22	Specific intervention(s) in the intensive care unit: endotracheal intubation, insertion of pacemaker, cardioversion, endoscopies, emergency surgery in the previous 24 h, gastric lavage; routine interventions without direct consequences to the clinical condition of the patient, such as radiographs, echography, electrocardiogram, dressings, or insertion of venous or arterial catheters, are not included	2.8
23	Specific interventions outside the intensive care unit: surgery or diagnostic procedures	1.9
